# A single sequence MRI-based deep learning radiomics model in the diagnosis of early osteonecrosis of femoral head

**DOI:** 10.3389/fbioe.2024.1471692

**Published:** 2024-08-30

**Authors:** Tariq Alkhatatbeh, Ahmad Alkhatatbeh, Xiaohui Li, Wei Wang

**Affiliations:** ^1^ Comprehensive Orthopedic Surgery Department, The Second Affiliated Hospital of Xi’an Jiaotong University, Xi’an, China; ^2^ Department of Orthopedics, The First Affiliated Hospital of Shantou University Medical College, Shantou, China; ^3^ Department of Radiology, The Second Affiliated Hospital of Xi’an Jiaotong University, Xi’an, China

**Keywords:** radiomics, deep learning, osteonecrosis, femoral head, magnetic resonance image

## Abstract

**Purpose:**

The objective of this study was to create and assess a Deep Learning-Based Radiomics model using a single sequence MRI that could accurately predict early Femoral Head Osteonecrosis (ONFH). This is the first time such a model was used for the diagnosis of early ONFH. Its simpler than the previously published multi-sequence MRI radiomics based method, and it implements Deep learning to improve on radiomics. It has the potential to be highly beneficial in the early stages of diagnosis and treatment planning.

**Methods:**

MRI scans from 150 patients in total (80 healthy, 70 necrotic) were used, and split into training and testing sets in a 7:3 ratio. Handcrafted as well as deep learning features were retrieved from Tesla 2 weighted (T2W1) MRI slices. After a rigorous selection process, these features were used to construct three models: a Radiomics-based (Rad-model), a Deep Learning-based (DL-model), and a Deep Learning-based Radiomics (DLR-model). The performance of these models in predicting early ONFH was evaluated by comparing them using the receiver operating characteristic (ROC) and decision curve analysis (DCA).

**Results:**

1,197 handcrafted radiomics and 512 DL features were extracted then processed; after the final selection: 15 features were used for the Rad-model, 12 features for the DL-model, and only 9 features were selected for the DLR-model. The most effective algorithm that was used in all of the models was Logistic regression (LR). The Rad-model depicted good results outperforming the DL-model; AUC = 0.944 (95%CI, 0.862–1.000) and AUC = 0.930 (95%CI, 0.838–1.000) respectively. The DLR-model showed superior results to both Rad-model and the DL-model; AUC = 0.968 (95%CI, 0.909–1.000); and a sensitivity of 0.95 and specificity of 0.920. The DCA showed that DLR had a greater net clinical benefit in detecting early ONFH.

**Conclusion:**

Using a single sequence MRI scan, our work constructed and verified a Deep Learning-Based Radiomics Model for early ONFH diagnosis. This strategy outperformed a Deep learning technique based on Resnet18 and a model based on Radiomics. This straightforward method can offer essential diagnostic data promptly and enhance early therapy strategizing for individuals with ONFH, all while utilizing just one MRI sequence and a more standardized and objective interpretation of MRI images.

## 1 Introduction

Osteonecrosis of the femoral head (ONFH) is a very common pathology of the hip that can lead to activity restrictions and catastrophic lifestyle changes. As demonstrated in our previous publication (Hu et al., 2024), the prevalence and the incidence of ONFH have significantly increased and may continue to increase, at least in China, for the next 2 decades. Therefore, early diagnosis and treatment planning of ONFH is crucial to preserve the femoral head and improve the prognosis. In 2019, The Association Research Circulation Osseous Staging System (ARCO) published a revised classification version for ONFH. They classified ONFH into Stage I: X-ray results are within normal range, but either magnetic resonance imaging (MRI) or scans of bones show positive findings. Stage II: The X-ray shows aberrant findings such as minor signals of increased bone density, localized bone loss, or cystic changes in the femoral head. However, there is no evidence of a fracture in the underlying bone, fracture in the necrotic area, or flattening of the femoral head. Stage III refers to a fracture that occurs in the subchondral or necrotic zone, which can be observed on X-ray or computed tomography (CT) scans. The third stage was subdivided into two categories: stage IIIA, which refers to early femoral head depression of 2 mm or less, and stage IIIB, which refers to late femoral head depression of more than 2 mm. Additionally, stage IV indicates the presence of osteoarthritis as evidenced by X-ray findings such as joint space narrowing, acetabular alterations, and/or joint destruction (Yoon et al., 2020). Our study and, according to the revised ARCO classification, classified only Stage I and II as early ONFH; afterwards we made use of their MRI scans for this investigation.

Traditional imaging modalities such as X-ray and CT (computed tomography) have restrictions when it comes to identifying early stages of osteonecrosis of the femoral head (ONFH), as there are no visible bone changes at this stage using these techniques. Therefore, the widely accepted gold standard for diagnosing early ONFH is MRI (Hu et al., 2015). However, it is user-dependent, which makes the interpretation of MRI vary between different radiologists. Thus, this necessitates the need for a robust and standard, reliable way to detect early ONFH using MRI.

Deep learning showed some promising results and an outstanding performance in detecting and classifying ONFH (Shen et al., 2023; Wang et al., 2021; Shen et al., 2024). But it comes at the cost of heavy data labeling time and preparations to train a successful model. Hence, making us look for another way that could achieve similar or better results with less work load.

Radiomics is a developing technique that entails converting regular radiological pictures into radiomics features. (Gillies et al., 2016), then recognizing important characteristics to create a distinctive framework for predicting clinical labels or outcomes. It is been widely used for the detection of various oncological changes by many researchers (Ding et al., 2021). In musculoskeletal disorders, Klontzas demonstrated that radiomics is capable of differentiating between Osteoporosis and Avascular necrosis (Klontzas et al., 2021). Wang introduced a radiomics method that utilizes MRI scans to diagnose early ONFH. His method specifically makes use of Multi-sequence MRI. In his investigation, he utilized T1 weighted with fat suppression and T2 weighted radiographs, along with coronal short time of inversion recovery pictures (Cor STIR) (Wang et al., 2024). Cheng reported that using a combined model of both Deep Learning and Radiomics; displayed an outstanding ability in diagnosing osteoporosis (Cheng et al., 2023). Liu also reported similar findings when combining radiomics with deep learning, he utilized Boruta selection to find the key features; and was able to distinguish between glioblastoma and brain metastasis (Liu et al., 2021). Another paper applied both deep learning and radiomics to differentiate between brain abscess and cystic glioma (Bo et al., 2021); they analyzed the features by spearman rank correlation test. Our aim is to be able to precisely diagnose early ONFH using a single-sequence MRI while reducing the up-front workload and the complexity of using different MRI sequences. Therefore, and for the first time; we are utilizing a Deep Learning-based Radiomics model for this purpose.

## 2 Materials and methods

### 2.1 Study participants

**FIGURE 1:**
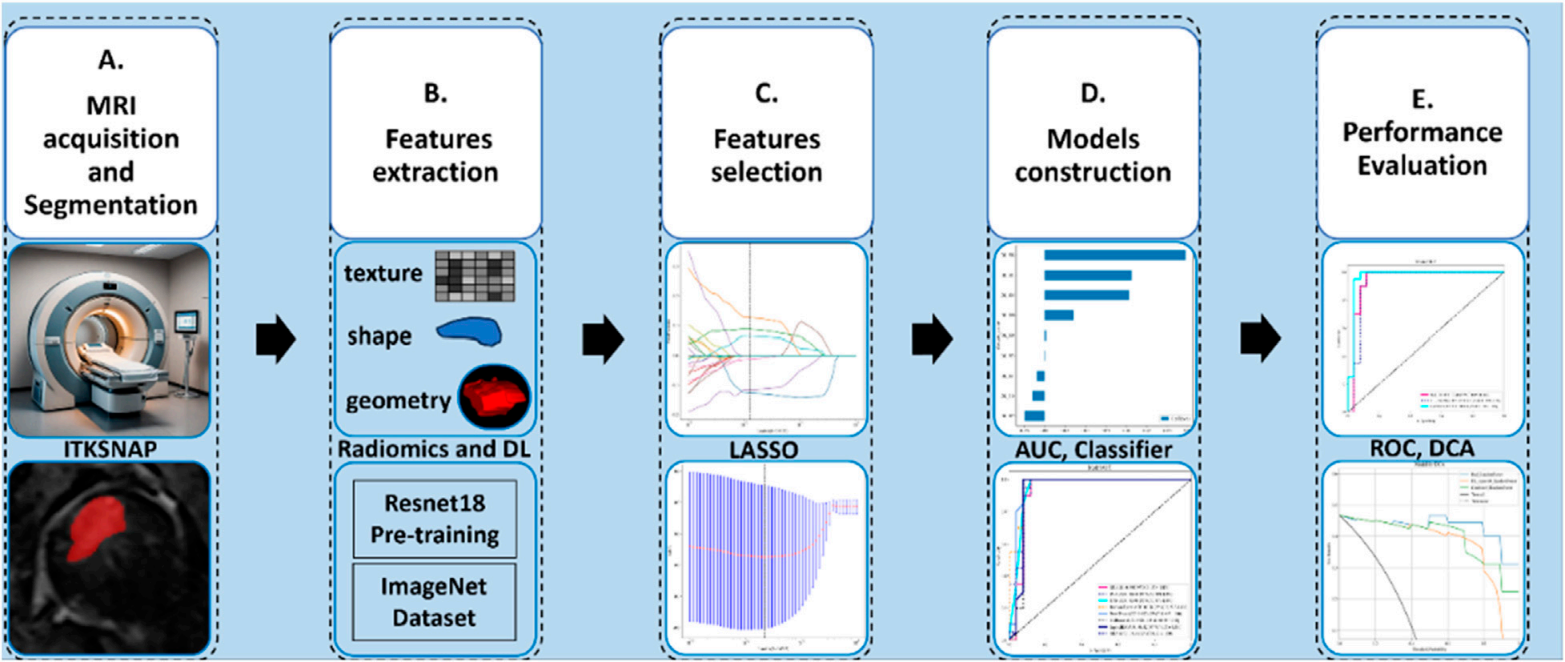
This study’s design and work pipeline.

A total of 150 patients were included in this retrospective research: 80 healthy patients MRIs were acquired from those who came for routine checkups without any clinical or radiologic signs and symptoms of ONFH. In addition, 70 patients’ radiographs were diagnosed by the radiology department of Xi’an Jiaotong University Second Hospital with early ONFH between FEB 2016 and APR 2024. The eligibility criteria for the patients were: 1) exhibited clinical symptoms such as hip pain or activity restriction. 2) only stage 1 and 2 ONFH patients were included based on ARCO classification. 3) Positive, clear MRI without artifacts. 4) No evidence of femoral head depression or fracture on normal radiograph, as that indicates a later stage of the disease. The data was acquired from the digital health record system of our hospital. The study design pipeline is illustrated in Figure 1. This retrospective investigation obtained permission from the ethics oversight Board of our hospital, with no requirement of informed consent from patients.

### 2.2 Region of interest segmentation

The MRI images were taken using a (Avanto, Siemens Healthineers; Erlangen, Germany) 1.5 T (tesla) scanner with the following values: Sequence type turbo spin echo (TSE); T2-weighted with fat suppressed (FS); slice thickness 4.5 mm, FOV 640*640 mm, acquisition matrix 0\320\240\, echo time 67 ms, repetition time 3000 ms, in the coronal plane; (Headfirst- Supine) position.

One skilled orthopedic surgeon (5 years of experience) manually segmented the region of interest (ROI) on each MRI slice; using ITK-SNAP 4.2.0 (https://www.itksnap.org/). Subsequently, an imaging specialist with 6 years of expertise in interpreting musculoskeletal MRI scans carefully examined all the segmented pictures to confirm their accuracy. Any disagreements were discussed or fixed till a satisfactory result was obtained. The ROI for healthy patients consisted of the femoral head and the closer part of the femoral neck. However, for patients with ONFH, the ROI was limited to the necrotic area only.

### 2.3 Image preprocessing

The cases were split into training and testing groups using a random distribution, with a ratio of 7:3. The entire training dataset was utilized to train the predictive model, whereas instances in the testing dataset were employed for the internal evaluation of the models’ performance. In this experiment, we employed the fixed-resolution resampling method to address any variations in voxel spacing. The voxel spacing of all pictures was standardized by resampling them to a size of 1*1*1 mm. Ultimately, the data underwent z-score standardization, also known as zero-mean normalization.

### 2.4 Features extraction

The feature extraction in this work included conventional handcrafted radiomic features derived from the original radiographs, such as geometry, intensity, and texture. In addition to the deep learning features derived automatically from a Convolutional Neural Network (CNN) utilizing training data.

PyRadiomics was utilized to extract radiomic features. The manually generated radiomic features can be classified into three distinct categories: (a) geometric, (b) intensity, and (c) textural. The shape in three dimensions of the necrotic cells is referred to by the geometry features. The intensity features analyze the statistical spread of voxel intensities within the femoral head using first-order statistics. The texture features indicate the features that describe the patterns or the spatial distributions of intensities beyond the first order. Different approaches, including the gray-level run length matrix (GLRLM), neighborhood gray-tone difference matrix (NGTDM), gray-level size zone matrix (GLSZM), and the gray-level co-occurrence matrix (GLCM) are employed to retrieve texture features. Furthermore, to achieve high-throughput features, the nonlinear intensity of image voxels is converted using various transformations such as Square, Square Root, Logarithm, Gradient, LBP3D, and Exponential. The high Laplace filter utilizes sigma values of 1, 2, and 3. Additionally, the process of extracting first-order statistics and texture features involved the use of eight wavelet transform algorithms: HLL, HLH, HHL, HHH, LHH, LLL, LLH, and LHL. For a comprehensive explanation of all image features, please refer to the online resource at https://pyradiomics.readthedocs.io/en/latest/features.html.

The Resnet18 model was used as the convolutional neural network (CNN) architecture in this investigation to obtain deep learning features. The MRI slice with the largest necrotic area was cropped and chosen for each patient. Hence, the network was optimized by applying the stochastic gradient descent method. The Resnet18 model underwent pre-training using the ImageNet dataset (http://www.image-net.org/). Subsequently, the pre-trained model was utilized to initialize feature extraction. The model selected an average pooling layer to extract deep features and then used a principal component analysis to compress and obtain the finalized deep features. The CNN training parameters were as follows: batch size = 96, epochs = 30, and unit learning rate = 0.001.

### 2.5 Feature selection

Before feature selection, all the features were normalized by applying the z-score standardization method approach. Both the Radiomics and Deep Learning features underwent filtration through a series of four phases. 1) The Mann–Whitney U test was performed on all features, and only the features with a *P*-value less than 0.05 were retained. The Pearson test was employed to assess the relation between features and categories. Features having a *P*-value less than 0.05 were deemed possibly predictive. The Max-Relevance and Min-Redundancy (mRMR) technique was employed in our study, and it is been widely used in different radiomics methods before (Xie et al., 2024) to enhance the visualization of features by maximizing relevance and minimizing redundancy. Ultimately, the crucial features were evaluated by the utilization of the least absolute shrinkage and selection operators (LASSO).

### 2.6 Radiomics and deep learning (DL) models construction

Once the features were selected using LASSO, we utilized these features in a range of machine learning classifiers such as Random Forests (RF), K-Nearest Neighbors (KNN), Logistic Regression (LR), Support Vector Machines (SVM), XGBoost, and others. After comparing all the parameters, we chose the highest performance to build the prediction models. Here, we utilized 5-fold cross-validation to build the final Rad and DL models. To evaluate whether a combination of the features mentioned above could produce superior outcomes. Using the previously described approach, a Deep Learning-Based Radiomics (DLR) model was built by combining features from both Deep Learning and Radiomics.

### 2.7 Statistical analysis

The evaluation of data was conducted using the Python Statsmodels package (0.13.2 version), and a *p*-value below 0.05 was considered to have statistical significance. The predictive models’ clinical importance in diagnosing early ONFH was evaluated by plotting Receiver Operating Characteristic (ROC) curves and analyzing the corresponding Area Under the Curve (AUC), diagnostic accuracy, sensitivity, specificity, positive predictive value (PPV), and negative predictive value (NPV). In addition, the model’s discriminative power was assessed using calibration curves and decision curve analysis (DCA). We also utilized Delong’s test to compare the ROC curve AUCs.

## 3 Results

### 3.1 Patients’ characteristics

The study comprised a total of 150 patients’ MRI scans, consisting of 80 healthy individuals and 70 patients with early ONFH, based on the specified criteria for inclusion. The patients were categorized into a training group of 105 individuals and a testing group of 45 individuals. Table 1 demonstrates a summary of the patient’s primary attributes.

**TABLE 1:** Basic characteristics of a total of 150 patients.

Characteristic	Healthy patients (*n* = 80)	Early ONFH patients (*n* = 70)
Age (years) Mean ± SD	40.1 ± 14.04	47.21 ± 14.91
Gender, No. (%)		
Male	30 (37.5%)	34 (48.58%)
Female	50 (62.5%)	36 (51.42%)

### 3.2 Feature extraction and selection

By utilizing a special feature analysis software integrated into Pyradiomics (http://pyradiomics.readthedocs.io), a total of 1,197 Radiomics features have been retrieved. The features included: 234 First Order, 182 (GLDM), 208 (GLRLM), 208 (GLSZM), 65 (NGTDM), 286 (GLCM), and 14 Shape features. Furthermore, Resnet18, which was pre-trained using the slice with the most necrotic tissue in its cross-section, was used to extract a total of 512 DL features.

We conducted a Mann-Whitney U test and performed feature screening on all of the chosen features. Only features with a *P*-value less than 0.05 were retained, resulting in the following numbers: The Rad model consists of 983 features. The DL model consists of 487 characteristics. The DLR model consists of 1,534 features.

The second phase involved evaluating features with high repeatability by utilizing the Pearson correlation coefficient, which measures the correlation between features. If the correlation coefficient between any two features exceeded 0.9, only one of them was kept. The Rad-model had 195 features, the DL-model had 35 features, and the DLR-model had 244 features.

In the third step, to ensure maximum feature representation, Max-Relevance and Min-Redundancy (mRMR) were used for further feature filtering. Rad-model = 30, DL-model = 30, DLR-model = 30 features.

**FIGURE 2:**
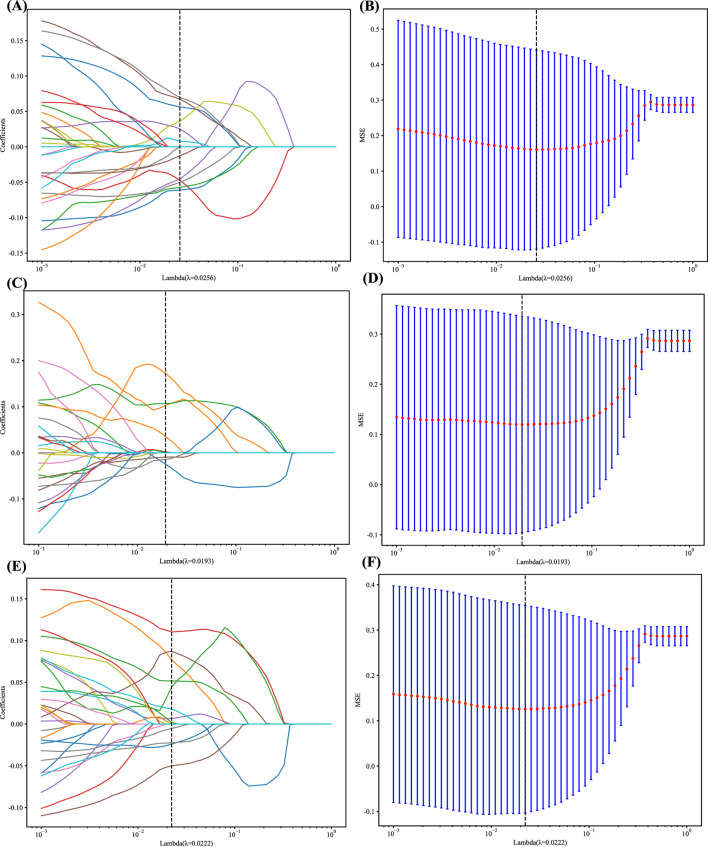
**(A, C and E)** LASSO Coefficients profile plot with various log(λ) is displayed; the vertical dashed line represents the selected features with nonzero coefficients chosen to the optimal lambda **(B, D and F)** MSE of 10-fold cross-validation for the most valuable features screened for the Rad, DL, and DLR models, respectively.

**FIGURE 3:**
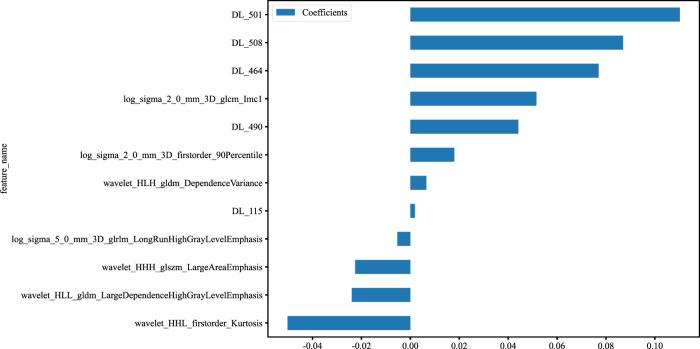
Histogram of the features selected for the DLR model, displaying each feature’s contribution.

In addition, the logistic regression model (LASSO) was employed to minimize the number of features and identify the most significant features for constructing the model. LASSO applies a shrinkage technique to all regression coefficients, pushing them toward zero and specifically setting the coefficients of unimportant features to zero based on the regulation weight Lambda (λ). In order to determine the ideal value of λ, a 10-fold cross-validation was conducted using a minimal criteria approach. The value of λ that resulted in the lowest cross-validation error was selected as the final value. The kept features with non-zero coefficients were utilized to fit a regression model and then integrated to create a Radiomics model. Afterward, we calculated a radiomics score for each patient by multiplying the retained features with their respective model coefficients and summing them up. The LASSO regression modeling was performed using the Python scikit-learn package, identifying 12 radiomics features, 14 DL features, and 9 DLR features. The figures below display the mean square errors (MSE) obtained from 10-fold validation, as well as a coefficient profile plot of the LASSO models. Each curve in the plot depicts the changing trajectory of each independent predictor. Figures 2A, C, E Explains the process of feature selection using the least absolute shrinkage and selection operator (LASSO) logistic regression in the Radiomics-model (A), DL-model (C), DLR-model (E). Figure 2 Shows the mean squared error (MSE) values obtained from doing 10-fold cross-validation for Radiomics-model (B), DL-model (D), DLR-model (F). The histogram of the selected features for the DLR-model is displayed in Figure 3.

### 3.3 Predictive performance of radiomics, DL, and DLR models

**FIGURE 4:**
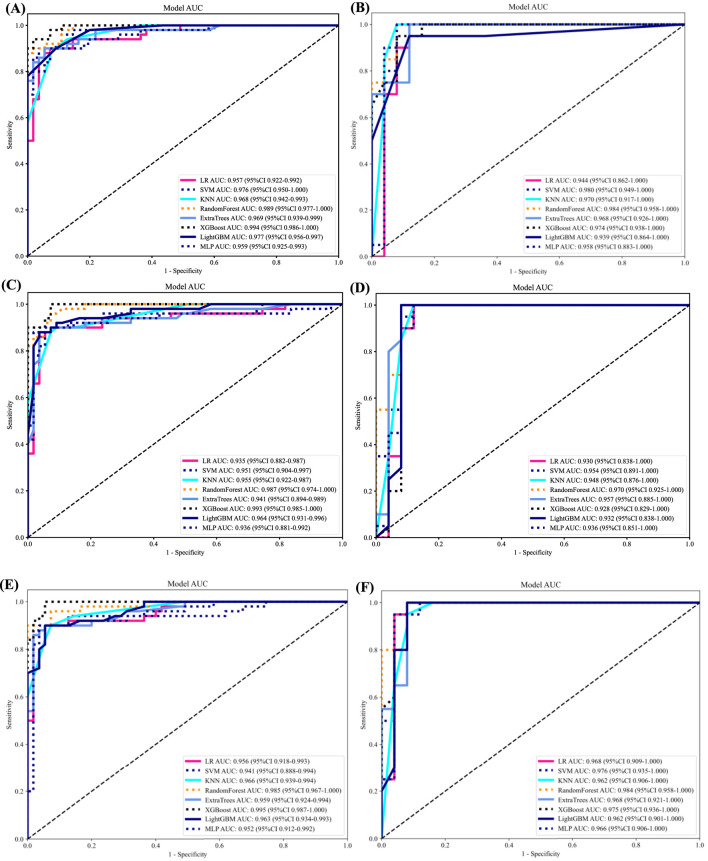
ROC curves for all models in both the training and testing groups. **(A)** ROC curves of different classifiers on Radiomic-model (training). **(B)** ROC curves of different classifiers on Radiomic-model (testing) **(C)** ROC curves of different classifiers on DL-model (training). **(D)** ROC curves of different classifiers on Radiomic-model (testing). **(E)** ROC curves of different classifiers on DLR-model (training). **(F)** ROC curves of different classifiers on DLR-model (testing).

As LR performed almost the best in each model, it was the classifier of choice for the construction of the Rad, DL, and DLR models. The Rad-model showed good results with an (AUC = 0.957) and (AUC = 0.944) in both the training and testing cohort, respectively, as shown in Figure 4 training (A), testing (B). Which outperformed the DL-model that showed (AUC = 0.935) and (AUC = 0.930) for the training and testing cohort correspondingly; illustrated in Figure 4 training (C), testing (D).

**FIGURE 5:**
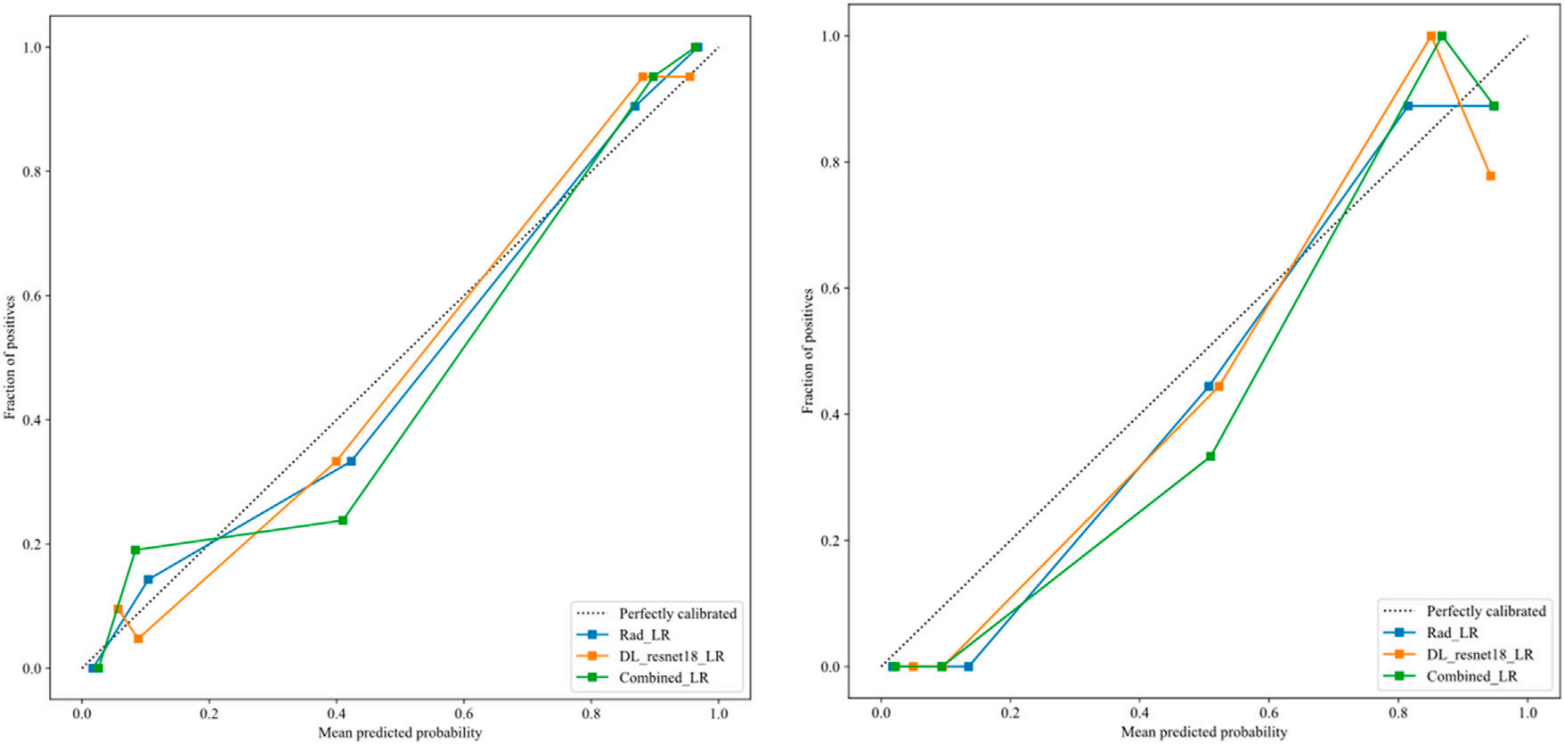
The calibration curves for DLR-model in the training group (left), and testing group (right), demonstrate a strong correlation between the average predicted probability (*x*-axis) and the proportion of positive outcomes (*y*-axis), indicating successful calibration with the perfect calibrated line.

**FIGURE 6:**
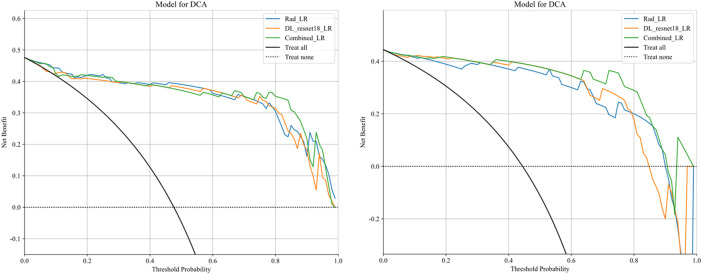
Decision curve analysis was performed on the DLR-model across the training group (left) and the testing group (right). The *y*-axis represents the net benefit, while the *x*-axis represents the threshold probability. The DLR model demonstrates a superior total beneficial effect in predicting early ONFH in healthy patients.

After the fusion of the selected Radiomics and DL features, the DLR-model was developed by integrating all (Kocak et al., 2024) features together in one model. The significant features selected for the DLR-model were as follows: DLR_LR_model = −0.2678082691039214 + +0.440703 * DL_464 + 0.444099 * log_sigma_2_0_mm_3D_glcm_Imc1 +0.576826 * DL_501–0.026022 * wavelet_HLH_gldm_DependenceVariance −0.590691 * wavelet_HHL_firstorder_Kurtosis +0.253499 * log_sigma_2_0_mm_3D_firstorder_90Percentile +0.377429 * DL_115–0.238724 * log_sigma_5_0_mm_3D_glrlm_LongRunHighGrayLevelEmphasis −0.656008 * wavelet_HLL_gldm_LargeDependenceHighGrayLevelEmphasis +0.089201 * DL_490 + 0.337023 * DL_508–0.160717 * wavelet_HHH_glszm_LargeAreaEmphasis. An improved performance over both the Rad-model and DL-model was found for the DLR-model; (AUC = 0.956) in the training dataset and (AUC = 0.968) in the testing dataset as displayed in Figure 4 training (E), testing (F). The diagnostic AUC, 95%CI, accuracy, sensitivity, specificity, PPV, NPV, precision, recall, and F1 of the three models are likewise demonstrated in Table 2. In addition, the calibration curves showed good agreement between all models, as shown in Figure 5. The *P*-values of the Hosmer-LemeShow test in Table 3 were 0.446, 0.051, and 0.234 for the Radiomics, DL, and DLR models, respectively. This indicates a good-fitting model, as all of the values were greater than 0.05. Delong’s test has been used to compare the ROC curve AUCs of all models, as shown in Table 4. Both the CLEAR (Kocak et al., 2023) and METRICS (Kocak et al., 2024) checklists of this study were presented in Supplementary Figures S1, S2. Furthermore, the net benefit was plotted against threshold probability in Figure 6, which displays the Decision curve analysis (DCA); it indicates that the DLR-model has the highest net benefit in identifying ONFH, which means that the DLR-model was useful for predicting early ONFH from healthy patients.

**TABLE 2:** All the metrics for the Radiomic, DL, and DLR models.

Model	Classifier	Acc	AUC	95% CI	Sen	Spec	PPV	NPV	PREC	Recall	F1	Cohort
Rad	LR	0.905	0.957	0.9217–0.9925	0.88	0.927	0.917	0.895	0.917	0.88	0.898	Train
Rad	LR	0.911	0.944	0.8622–1.0000	0.95	0.880	0.864	0.957	0.864	0.95	0.905	Test
DL	LR	0.905	0.935	0.8824–0.9874	0.88	0.927	0.917	0.895	0.917	0.88	0.898	Train
DL	LR	0.911	0.930	0.8377–1.0000	0.95	0.880	0.864	0.957	0.864	0.95	0.905	Test
DLR	LR	0.914	0.956	0.9183–0.9930	0.88	0.945	0.936	0.897	0.936	0.88	0.907	Train
DLR	LR	0.933	0.968	0.9085–1.0000	0.95	0.920	0.905	0.958	0.905	0.95	0.927	Test

ACC, Accuracy; AUC, area under curve; CI, confidence interval; SEN, Sensitivity; Spec, Specificity; PPV, positive predictive value; NPV, negative predictive value; PREC, Precision.

**TABLE 3:** Illustrates the significance levels (*p* values) obtained by the Hosmer-Lemeshow test, which is used to assess the goodness-of-fit of models.

Model	Rad-model	DL-model	DLR-model	Cohort
P	0.952	0.549	0.380	Train
P	0.446	0.051	0.234	Test

**TABLE 4:** Delong test for each of the models.

Cohort	DLR Vs. Rad	DLR Vs. DL	DL Vs. Rad
Train	0.870	0.133	0.185
Test	0.594	0.145	0.800

## 4 Discussion

**FIGURE 7:**
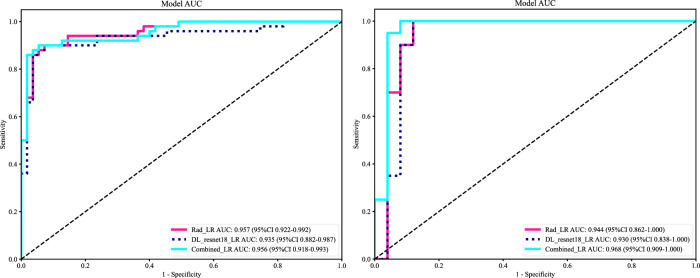
ROC of the radiomic-model, DL-model, and DLR-model, training set (left), testing set (right). That shows an improvement of AUC = 0.968 using the fused features.

In this study, we presented how Radiomics and Deep learning features can be combined to make a Deep Learning-based Radiomics model that can predict early ONFH accurately; AUC = 0.968 (95%CI 0.909–1.000). This model has shown superior results to both the Rad-model (AUC = 0.944 (95%CI 0.862–1.000) and the DL-model (AUC = 0.930 (95%CI 0.838–1.000). The ROC for the three models is illustrated in Figure 7; it shows that the DLR-model has an improved and higher AUC = 0.968 than the other two models. Our study was based on a single sequence MRI (T2W1), that has been utilized to extract Radiomics and Deep learning features. Unlike Wang (Wang et al., 2024), which used a multi-sequence (T1W1, FS-T2W1, and Cor STIR) MRI-based method to predict early ONFH using Radiomics only. He claimed that in order to thoroughly diagnose early ONFH, it is imperative to take into account various MRI sequences. However, we have demonstrated that a single sequence MRI is sufficient and can achieve a high accuracy using a Deep Learning-based Radiomics method rather than multi-MRI sequences and Radiomics only. In his study, he also considered ONFH stage I, II and IIIA as early stages, which we disagree with. According to the revised ARCO classification (Yoon et al., 2020), only stage I and II are considered early stages. Stage IIIA shows microfractures or depression in the femoral head that could be visible on a more affordable CT scan and does not necessarily need an MRI that is more expensive and might not be available in many institutions. In addition, the whole purpose of using radiomics or deep learning in detecting early ONFH is accuracy and simplicity. Hence, achieving those results using a single widely used MRI sequence is a great advantage. Besides that, Klontazs applied radiomics and machine learning to differentiate between Osteoporosis and avascular necrosis of the hip (AVN). It was not mentioned what stages were included or excluded or following which grading system for AVN. Following feature extraction, he only used three machine learning classifiers (XGboost, CatBoost and SVM) to perform the experiment. Whereas we have used LR, SVM, KNN, RandomForest, ExtraTrees, XGBoost, LightGBM, and MLP and compared them all to obtain the best performing model. In his paper, XGboost displayed the best results achieving AUC of 93.7%. on the other hand, our top performer LR based on the combined model; showed superior results with an AUC of 0.9698. Other previous studies have used deep-learning methods to detect early ONFH (Shen et al., 2023; Wang et al., 2021; Klontzas et al., 2023; Li et al., 2023). Deep learning uses features from a single image with the largest necrotic area, whereas radiomics obtains quantitative features from multiple MRI slices at once. It can detect more features, as we proved in our study; we could extract 1,197 radiomic features, whereas deep learning features were 512 only. In addition, deep learning requires an extensive amount of labeled data for the training. Nonetheless, radiomics has an advantage over DL as it is effective even when using smaller datasets. We have not compared our results to a radiologist, which is a disadvantage of our study. Still, other studies did compare them, and radiomics always had either similar results to an experienced radiologist or even better ones (Klontzas et al., 2021; Wang et al., 2024). We think by using a combined Deep Learning-Based Radiomics method and a single sequence MRI only; we provided a great diagnostic method for the early detection of ONFH and a significant contribution to the research. As far as we know, we are the first to combine deep learning and radiomics for this specific task. Our study has some limitations, including 1) moderate sample size for both the training and testing. 2) We have made our study only using a single center, a multi-center study in the future could further display better analysis for using Radiomics to detect early ONFH. In conclusion, using a single sequence MRI scan, our work constructed and verified a Deep Learning-Based Radiomics Model for early ONFH diagnosis. This strategy outperformed a Deep learning technique based on Resnet18 and a model based on Radiomics. This straightforward method can offer essential diagnostic data promptly and enhance early therapy strategizing for individuals with ONFH, all while utilizing just one MRI sequence and a more standardized and objective interpretation of MRI images.

## 5 Conclusion

In conclusion, using a single sequence MRI scan, our work constructed and verified a Deep Learning-Based Radiomics Model for early ONFH diagnosis. This strategy outperformed a Deep learning technique based on Resnet18 and a model based on Radiomics. This straightforward method can offer essential diagnostic data promptly and enhance early therapy strategizing for individuals with ONFH, all while utilizing just one MRI sequence and a more standardized and objective interpretation of MRI images.

## Data Availability

The original contributions presented in the study are included in the article/Supplementary Material, further inquiries can be directed to the corresponding author.
